# Understanding the plant-pathogen interactions in the context of proteomics-generated apoplastic proteins inventory

**DOI:** 10.3389/fpls.2015.00352

**Published:** 2015-06-02

**Authors:** Ravi Gupta, So Eui Lee, Ganesh K. Agrawal, Randeep Rakwal, Sangryeol Park, Yiming Wang, Sun T. Kim

**Affiliations:** ^1^Department of Plant Bioscience, Life and Industry Convergence Research Institute, Pusan National UniversityMiryang, South Korea; ^2^Research Laboratory for Biotechnology and BiochemistryKathmandu, Nepal; ^3^Global Research Arch for Developing Education (GRADE), Academy Private LimitedBirgunj, Nepal; ^4^Organization for Educational Initiatives, University of TsukubaTsukuba, Japan; ^5^Faculty of Health and Sport Sciences, Tsukuba International Academy for Sport Studies, University of TsukubaTsukuba, Japan; ^6^Bio-crop Development Division, National Academy of Agricultural Science, Rural Development AdministrationJeonju, South Korea; ^7^Department of Plant Microbe Interactions, Max Planck Institute for Plant Breeding ResearchCologne, Germany

**Keywords:** apoplast, apoplastic proteins, pattern-triggered immunity, effector-triggered immunity, secretome, protein secretion, plant-pathogen interaction

## Abstract

The extracellular space between cell wall and plasma membrane acts as the first battle field between plants and pathogens. Bacteria, fungi, and oomycetes that colonize the living plant tissues are encased in this narrow region in the initial step of infection. Therefore, the apoplastic region is believed to be an interface which mediates the first crosstalk between host and pathogen. The secreted proteins and other metabolites, derived from both host and pathogen, interact in this apoplastic region and govern the final relationship between them. Hence, investigation of protein secretion and apoplastic interaction could provide a better understanding of plant-microbe interaction. Here, we are briefly discussing the methods available for the isolation and normalization of the apoplastic proteins, as well as the current state of secretome studies focused on the *in-planta* interaction between the host and the pathogen.

## Introduction

Plant-pathogen interaction is a multifaceted process, mediated by the pathogen- and plant-derived molecules which mainly include proteins, sugars and lipopolysaccharides (Boyd et al., 2013). Secreted molecules, derived from the pathogens, are the key factors which determine their pathogenicity and allow their successful colonization inside the host. On the other hand, plant derived molecules are involved in the recognition of these pathogens in order to elicit the defense response. The first interaction between the plants and microbes take place in apoplast and is mediated by the recognition of microbial elicitors by the receptor proteins of the plants (Dodds and Rathjen, 2010). These microbial elicitors, also known as pathogen-associated molecular patterns (PAMPs), are recognized by the membrane-localized pattern recognition receptors (PRRs) of plants (Boyd et al., 2013; Zipfel, 2014). The bacterial flagellin and elongation factor (EF)-Tu peptide surrogates, flg22 and elf18, and chitin, are common examples of PAMPs, which are recognized by the plant PRRs that include the three receptor-like kinases, flagellin-sensitive22 (FLS2), EF-Tu receptor (EFR), and chitin elicitor receptor kinase1 (CERK1) (Liu et al., 2013). The successful recognition of microbial derived PAMPs by PRRs of the plants activates a first line of defense which is known as PAMP-triggered immunity (PTI). To counter-attack the PTI, many pathogens deliver various “effector” proteins inside the host cell, which suppress the components of PTI. These pathogen derived “effector” proteins include various avirulence (Avr) proteins like Slp1 of *Magnoporthe oryzae* and TALEs of *Xanthomonas oryzae* (Boyd et al., 2013; Liu et al., 2014a). However, resistance (R) proteins of plants recognize these effector proteins of pathogens and can induce a second line of defense which is known as the effector-triggered immunity (ETI) (Jones and Dangl, 2006). ETI is quantitatively stronger and faster than PTI and can result in a localized cell death (hypersensitive response) to kill both pathogen and pathogen infected plant cells. PTI and ETI together constitute a major innate immune response, enabling plants to recognize and battle against the pathogen attack. However, the components of ETI and PTI in response to interaction with different pathogens remain largely unknown, requiring a large-scale investigation of proteins for better understanding of the plant-pathogen interactions, which would be important to generate the stress tolerant crops. As the first interaction between plant and pathogens occur in apoplast, analyzing the dynamic changes of apoplastic proteins through proteomics approach is necessary for a deep understanding of the components of signal perception and signal transduction during pathogen attack.

The past few years have seen remarkable efforts in solving the mystery of plant-pathogen interaction in the apoplast (reviewed in Krause et al., 2013; Delaunois et al., 2014; Tanveer et al., 2014). For the analysis of secreted proteins in response to pathogen attack, mostly *in-vitro* interaction systems using suspension-cultured cells were used, due to relatively easy isolation of secreted proteins from them (reviewed in, Agrawal et al., 2010). However, recent comparative studies strongly suggest that the components of the *in-vitro* and the *in-planta* secretome can be relatively different, sharing sometimes less than 3% of common proteins. A comparison of the *in-vitro* and *in-planta* secreted proteins showed only 6 common proteins out of the total 222 identified proteins in rice (Jung et al., 2008). Moreover, the *in-vitro* secretome analysis may not illustrate the real state of host-pathogen interaction, thereby necessitating extraction of apoplastic proteins from the *in-planta* systems. In this mini-review, we have summarized the progress made so far in this area to present the current scenario of secretomics during the plant-pathogen interaction.

## Methods to isolate apoplastic proteins

**Figure 1:**
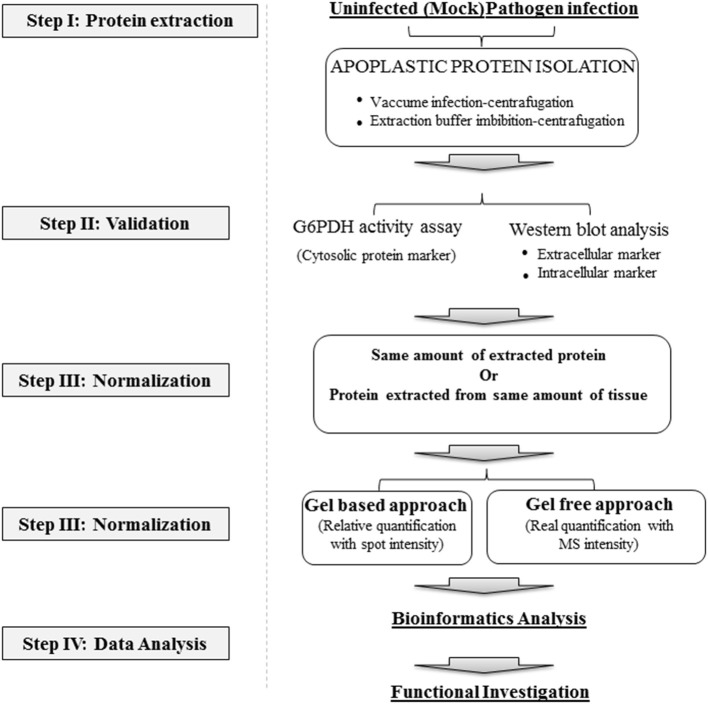
**Experimental strategy of the** ***in-planta*** **secretome studies during the host-pathogen interactions**. Details are in the text.

Due to the biochemical and technical advances, it is possible to isolate the proteins directly from the apoplast which can be analyzed by gel-based or gel-free proteomics approaches. However, relatively limited number of studies have been conducted so far, to identify the pathogen-secreted proteins *in-planta* (Table 1). The successful isolation of apoplastic proteins is the most critical step prior to utilizing the samples for proteome analysis. For the isolation of apoplastic proteins, a number of methods including vacuum infiltration (VIC) and gravity extraction methods are available (reviewed in Agrawal et al., 2010). However, only VIC method along with its modified version (termed CA-VIC), has been used for the isolation of apoplastic proteins in response to pathogen infection (Floerl et al., 2008) (Figure 1). In the VIC method, leaves are cut into small sections followed by extensive washings of these sections to remove cytoplasmic proteins from the cut ends. The washed leaves sections are then incubated in the extraction buffer which is allowed to infiltrate into the cells through a pressure change induced by vacuum. Finally, apoplastic proteins are recovered by centrifugation at low speed. This method was used to isolate the apoplastic proteins from the leaves of Arabidopsis and tobacco (De-la-Pena et al., 2008; Delannoy et al., 2008). However, this VIC method is less efficient in isolating the apoplastic proteins from the waxy coated leaves, like leaves of rice and maize. Moreover, previous studies in which apoplastic proteins were extracted from Arabidopsis and *Brassica* leaves by VIC method, showed identification of only few differential proteins in response to *Verticillium longisporum* infection, indicating the limitation of this method for comparative proteome analysis (Floerl et al., 2008: Floerl et al., 2012; Shenton et al., 2012). Furthermore, this VIC method yields much lower amount of apoplastic proteins which is a major constrain for large scale proteome analysis. Keeping these limitations in mind, the VIC method was modified (CA-VIC) to isolate the apoplastic proteins from rice leaves with increased amount (Figure 1). This method involves shaking of the cut segments of the leaves in a calcium based buffer for 1 h on ice, followed by vacuum infiltration, centrifugation, and phenol precipitation (Kim et al., 2013). This method yields higher amount of apoplastic proteins than classical VIC method, may be due to the addition of calcium, which facilitates the extraction of loosely bound cell wall proteins (Watson et al., 2004). A comparative analysis was carried out to select the best buffer for isolation of apoplastic proteins. Among all the extraction buffers tested, sodium phosphate or ascorbic acid with calcium chloride were the most efficient, while extraction with water or Tris showed contamination from vacuole and other organelles (Witzel et al., 2011; Gupta and Deswal, 2012). Therefore, the selection of an appropriate extraction method is crucial for apoplastic protein extraction in different plant species.

**Table 1:** **List of published** ***in-planta*** **secretome studies on plant-microbe interactions**.

**Plant**	**Pathogen**	**Apoplastic proteins isolation method**	**Identification method**	**References**
*Brassica napus*	*Verticillium longisporum*	Vacuum infiltration, centrifugation	2D-PAGE and ESI-LC-MS/MS	Floerl et al., 2008
*Arabidopsis thaliana*	*Verticillium longisporum*	Vacuum infiltration, centrifugation	2D-PAGE and ESI-LC-MS/MS	Floerl et al., 2012
*Oryzae sativa*	*Magnaporthe oryzae*	Vacuum infiltration, centrifugation	2D-PAGE and ESI-LC-MS/MS	Shenton et al., 2012
*Oryzae sativa*	*Magnaporthe oryzae*	Apoplastic buffer incubation, filtration	2D-PAGE/MudPIT and MALDI-TOF/MS or nESI-LC-MS/MS	Kim et al., 2013
*Oryzae sativa*	*Xanthomonas oryzae*	Apoplastic buffer incubation, filtration	2D-PAGE/MudPIT and MALDI-TOF/MS or nESI-LC-MS/MS	Wang et al., 2013
*Oryzae sativa*	*Cochliobolus miyabeanus*	Apoplastic buffer incubation, filtration	2D-PAGE/MudPIT and MALDI-TOFTOF/MS or nESI-LC-MS/MS	Kim et al., 2014

## Validation and normalization of apoplastic proteins

After isolation of apoplastic proteins, the next step is to assess the purity of isolated proteins as contamination from cytoplasm and other organelles can lead to the false positive results. To assess the purity of extracted apoplastic proteins, several methods including cytoplasmic marker enzymes activity assays and Western blot analysis of marker proteins, can be performed (Figure 1). Glucose-6-phosphate dehydrogenase (G6PDH), glyceraldehyde-3-phosphate dehydrogenase (GAPDH), and malate dehydrogenase (MDH) are some of the common cytoplasmic enzymes which are being widely used as biomarkers for cytoplasmic contamination, while RuBisCO antibodies are used for assessing the chloroplastic contamination in the apoplastic proteins. In addition to assessing the contamination, Western blots with antibodies against apoplastic markers like β-1,3-glucanase (Jung et al., 2008), Duf26 (or OsRMC) (Zhang et al., 2009), AtPR-1 (Wang et al., 2005), thaumatin-like protein and glucanase-2 (Kim et al., 2013), can also be used for sample validation to ensure the successful enrichment of apoplastic proteins. Moreover, this approach can also be employed to measure the contamination ratio with other cellular organelle markers. Previous reports have shown that both the vacuum infiltration and calcium buffer based apoplastic protein extraction methods show none/low ratio of contaminates (Agrawal et al., 2010; Gupta and Deswal, 2012; Kim et al., 2013). As an example, apoplastic proteins, isolated by CA-VIC method showed negligible cytoplasmic contamination as observed by low G6PDH activity. In addition, Western blots of intracellular proteins, OsPR-10 and PBZ1, did not detect any signal, suggesting the low levels of cytoplasmic contamination in isolated apoplastic proteins. Furthermore, the enrichment of apoplastic proteins was also shown by assessing the expression of apoplastic marker proteins (glucanse-2 and thaumatin-like protein) using Western blotting, indicating the efficacy of calcium based buffer in isolation of apoplastic proteins (Kim et al., 2013). Taken together, assessing the enrichment of apoplastic proteins and contaminations are the essential steps in the analysis of secretome.

To examine the differences in global protein secretion upon pathogen infection, sufficient normalization of protein samples is necessary. Based on previous studies, two possible normalization methods were applied to evaluate the protein abundances (Figure 1). In the first method, protein abundance is normalized with same amount of isolated proteins (Kaffarnik et al., 2009). As it is possible that the rate of protein secretion would also be affected in addition to changes in which proteins were secreted, normalization with protein concentration would provide absolute changes in protein identities (or proteins that changed dramatically in concentrations). However, as infection of pathogen could enhance the protein secretion in plants (Watanabe et al., 2013), and pathogen effectors could block the secretion of protein from plants (Lee et al., 2012), the disadvantage of normalization with protein amount is that the real protein secretion changes might be concealed. Another choice for sample normalization is on the fresh tissue amount. A significant increase of protein secretion was detected comparing with non-infected tissues (Kim et al., 2013). These results indicated that upon pathogen infection the overall protein secretion might be enhanced. Moreover, the selection of extraction buffer and protein loss during extraction procedures may strongly affect the final proteomics results. Therefore, normalization with fresh tissue amount may illustrate the real case of protein secretion upon pathogen infection. However, as it is difficult to distinguish the cytoplasmic and apoplastic proteins when normalized with the fresh tissue amount, use of cytoplasmic and apoplastic marker is highly recommended in order to check the cytoplasmic contamination. Taken together, the issue of how the amount of apoplastic proteins should be normalized has to be considered prior to the proteomics analysis.

## Proteomics investigations of in-planta secreted proteins during plant-pathogens interactions

**Figure 2:**
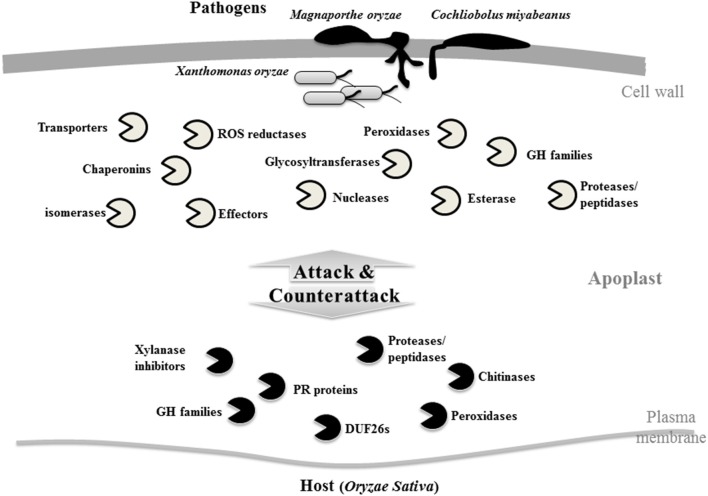
**Overview of the enriched apoplastic proteins identified from *in-planta* secretome studies during host-pathogen interactions**. High abundant secreted proteins derived from pathogens are listed on top while host secreted proteins are listed at the bottom.

*M. oryzae* (a hemi-biotrophic fungus) causes rice blast disease which results in huge loss of productivity of this most important cereal grain worldwide (Talbot, 2003). A gel-based proteomics approach was used to identify the rice-*M. oryzae* interaction in the apoplast which led to the identification of several rice secreted proteins including three DUF26 domain containing cysteine rich repeat proteins and PR-proteins. In addition, a *M. oryzae* secreted virulence factor protein, cyclophilin CYP1, was also identified in rice apoplast (Shenton et al., 2012). In another similar study, 732 secreted proteins were identified, of which 40 and 60% were from rice and *M. oryzae*, respectively (Kim et al., 2013). Furthermore, a higher level of up-regulation of glycosylhydrolase and chitinase proteins was observed in case of incompatible interactions as compared to the compatible, suggesting the involvement of these proteins in the resistance against rice blast fungus. These studies indicated that the pathogenic fungus also secretes numerous proteins into the apoplastic space. Plant secreted proteins were mainly glycosyl hydrolase family proteins, esterases, proteases and peptidases, suggesting that the cell wall and protein modifications are important aspects for the resistance against *M. oryzae* infection (Kim et al., 2013) (Figure 2). Identification of apoplastic proteins during rice-*Cochliobolus miyabeanus* (a necrotrophic fungus) infection led to the identification of 501 proteins, of which only 31 (6.2%) were secreted from *C. miyabeanus*, whereas 470 were secreted from rice. These results suggest that the host-secreted proteins are more abundant during the *C. miyabeanus* infection. Proteins with decreased abundance were mainly related to the Calvin cycle and glycolysis, whereas abundance of the proteins involved in the TCA cycle, amino acids, and ethylene biosynthesis was increased (Kim et al., 2014). *V. longisporum* is one of the most devastating diseases of the Brassicaceae members. Analysis of plant-*V. longisporum* interactions, using *Arabidopsis* and *Brassica* as hosts, showed up-regulation of various PR-proteins including endochitinase, peroxidases, β-1,3-glucanase, PR-4, PRX52, PRX34, P37, serine carboxypeptidase SCPL20, α-galactosidase AGAL2, and germin-like protein (Floerl et al., 2008, 2012). A lectin-like, chitin-inducible protein was down-regulated upon *V*. *longisporum* infection (Floerl et al., 2012). Surprisingly, no fungal protein was identified in these studies, suggesting that either the amount of fungal secreted proteins are much lower (beyond the detection limits of the MS) or *V. longisporum* majorly secretes other metabolites like sugars and lipopolysaccharides to interact with its hosts.

In addition to the fungal infections, apoplastic proteins were also analyzed during rice-bacterial infections. *X. oryzae* is a Gram-negative bacterium that causes the rice bacterial blight disease. Analysis of rice-*X. oryzae* interaction led to the identification of 109 proteins of which only six were secreted from rice, indicating that the percentage of bacterial-secreted proteins is much higher than its host rice (Wang et al., 2013). It was also shown that the highly conserved proteins secreted from *X. oryzae in-vitro* and *in-planta*, were related with the metabolic and nutrient uptake activities. The pathogenicity-related proteins were highly enriched *in-planta*, but not detected *in-vitro*, further implying differences in secretory proteins between *in-vitro* and *in-planta* systems. Overall, these findings suggest that the nutrient uptake from surrounding environments and sustaining basic metabolism is the primary task of fungus, while modification of host immunity is essential for *in-planta* survival and spread of the bacterium. Taken together, it can be concluded that the ratio of secretory proteins from host and pathogens (hemi-biotrophic, necrotrophic fungus, and Gram-negative bacteria) differs significantly, and likely their functions. However, it is worth mentioning here that the ratio of pathogen secreted proteins also varies in accordance with the compatible or incompatible interaction with the host. During the incompatible interactions, the growth of pathogens is very much reduced with much less secretion of protein while during compatible interactions the growth of pathogen is highly pronounced which results in secretion of more proteins (Gonzalez-Fernandez and Jorrin-Novo, 2012).

## Leaderless secretory proteins

Apoplastic proteins were long thought to be secreted only through the “Golgi-endoplasmic reticulum pathway” due to the presence of an N-terminal signal peptide. However, growing body of evidences suggest that the apoplast also harbors proteins which lack the signal peptide and therefore, these proteins are supposed to be secreted via non-classical protein secretion pathways (leaderless secretory pathways) (Agrawal et al., 2010; Kim et al., 2013; Wang et al., 2013). These proteins are well-known as the leaderless secretory proteins (LSPs) and constitute up to 80% of the total apoplastic proteins, depending upon the tissue and stress conditions (Agrawal et al., 2010). Metabolism-related proteins, which are mainly cytosolic, were commonly identified in the apoplastic region, suggesting that these proteins are pumped out through unknown mechanisms, and might be essential for plant immune responses. It is well documented that fungus secretes mannitol in the plant apoplastic space during infection to quench the reactive oxygen species which could otherwise elicit the plant defense response (Cheng and Williamson, 2010). On counter-attack, plants secret mannitol dehydrogenase (MTD) in the apoplast where it catabolizes the mannitol secreted by the fungus. However, MTD does not contain a signal peptide and therefore would be secreted by the non-classical pathways. Similarly, superoxide dismutase, which also lacks a signal peptide, has been confirmed as a resident of the apoplast (Cheng and Williamson, 2010; Gupta and Deswal, 2012). Many more such proteins have been identified and their inventory is increasing as we investigate more and further—thanks to the proteomics technologies.

In addition to these well characterized LSPs, several other cytoplasmic or non-secretory proteins are also observed in the apoplast during pathogen attack. These other cytoplasmic proteins might be the LSPs which are yet to be characterized. Notwithstanding, it can also be speculated that these proteins are just the contaminants which are released to the apoplast due to cell lysis. It is well known that both plant and pathogen secrete cell wall degrading enzymes which result in lyses of cell wall of opposite partner, thus resulting in the leakage of cytoplasmic proteins in the apoplast. However, there are few reports which neglect this possibility. During fungal infections, plants form extra-haustorial membrane (EHM) and extra-invasive hyphal membrane (EIHM), to separate the plant cytoplasm from the haustorium of oomycetes and invasive hypha of filamentous fungus respectively (Yi and Valent, 2013). These additional membranes maintain the integrity of the host cell and prevent its rapid lysis during pathogen infection, even though the cell wall is perforated. Due to the presence of these membranes, cytoplasmic proteins cannot be simply leaked out from the cytoplasm and therefore their accumulation in the apoplast must be mediated by some protein secretory pathway(s). Moreover, during the cell lysis, leakage of whole set of abundant cytoplasmic proteins is expected in the apoplast however, only limited set of cytoplasmic proteins are detected in the apoplast which further suggest controlled or regulated secretion of these proteins during pathogen attack (Kaffarnik et al., 2009).

## Prospects and further application

All the studies conducted so far to investigate the plant-microbe interactions have utilized a gel-based proteomics approach for the identification of *in-planta* secreted proteins. However, as gel based approaches have several limitations including identification of low-abundance proteins, it is possible that some of the key proteins of both plant as well as microbe, would be missed during the analysis. As an example, analysis of secreted proteins from *V. longisporum*-Arabidopsis or *Brassica* did not identify any fungal protein, may be due their low-abundance.

With the advancement in the proteomics technologies, new and more precise gel-free quantitative proteomics approaches with improved sensitivity are being developed which could be applied for protein identification, dynamic regulation, and analyzing the post-translational modifications (Picotti et al., 2009). Utilization of newly developed proteome tools like multiple reaction monitoring MS (MRM-MS), will benefit for the characterization and quantification of protein profile during plant-microbe interactions (Schumacher et al., 2014). Moreover, taking the advantage of genetic modified materials, such as plant or bacterial lacking secretion systems, much more novel proteins could be identified which can illustrate a deeper understanding of apoplastic interaction between host and pathogen (Hemsley et al., 2013; Schumacher et al., 2014). However, it still will be a long way to utilize those proteins for crop modification and further field applications. Fortunately, researchers have started looking inside the role of secreted proteins in plant-microbe interactions even only few publications were released. For instance, secretion of lysozyme-like hydrolase exhibits infections of bacterial pathogen by increasing the release of peptidoglycans from bacterial cell wall and triggering the PTI in Arabidopsis (Liu et al., 2014b). Those functional investigations of secreted proteins may provide better understanding of their role in plant-microbe interactions which would be helpful in the development of effective crop protection strategies (Gonzalez-Fernandez and Jorrin-Novo, 2012).

## Conclusions

Upon infection, both plants as well as pathogens secrete molecules including proteins which determine the fate of their interaction. While pathogen secreted proteins are involved in infection and pathogenicity, plant secreted proteins play crucial roles in its resistance. Therefore, identification and functional analyses of apoplastic proteins will open new horizons in our understanding of plant-pathogen interactions. Moreover, application of the *in-planta* protein extraction techniques at multiple times post-infection will reveal the “real” composition and dynamic changes of the apoplast and can result in the identification of more components of PTI. Furthermore, unraveling complete proteome of apoplastic region in multiple plants, pathogens, and their interactions would be highly fruitful for understanding the biology of plant-pathogen interactions, and that will help in designing new strategies for generating the next-generation crops resistance to multiple pathogens and environmental stresses.

### Conflict of interest statement

The authors declare that the research was conducted in the absence of any commercial or financial relationships that could be construed as a potential conflict of interest.
